# Overweight: A Protective Factor against Comorbidity in the Elderly

**DOI:** 10.3390/ijerph16193656

**Published:** 2019-09-29

**Authors:** Giovanni Mario Pes, Giulia Licheri, Sara Soro, Nunzio Pio Longo, Roberta Salis, Giulia Tomassini, Caterina Niolu, Alessandra Errigo, Maria Pina Dore

**Affiliations:** 1 Dipartimento di Scienze Mediche, Chirurgiche e Sperimentali, University of Sassari, 07100 Sassari, Italy; giulia.licheri@gmail.com (G.L.); sara.soro14@gmail.com (S.S.); nunziolongo@hotmail.it (N.P.L.); robertasalis@hotmail.it (R.S.); specialgastro2013@gmail.com (G.T.); caterina.niolu@hotmail.com (C.N.); mpdore@uniss.it (M.P.D.); 2 Sardinia Longevity Blue Zone Observatory, I-08040 Ogliastra, Italy; 3 Dipartimento di Scienze Biomediche, University of Sassari, 07100 Sassari, Italy; alessandra.errig@tiscali.it; 4 Baylor College of Medicine, One Baylor Plaza, Houston, TX 77030, USA

**Keywords:** Body height, comorbidity, Sardinia

## Abstract

The aim of this study was to investigate the relationship between body mass index (BMI) categories and comorbidity in 9067 patients (age range 18‒94 years) who underwent upper digestive endoscopy in Northern Sardinia, Italy. The majority of participants (62.2%) had a BMI under 25 kg/m^2^, overweight was detected in 30.4%, and obesity (BMI ≥ 30 kg/m^2^) in 7.4% of patients. The most frequent illness recorded was hypertension followed by cardiovascular and liver disease. The multivariate analysis, after adjusting for sex, residence, marital status, smoking habits, occupation and hospitalization detected an association between comorbidity and aging that was statistically significant and progressive. Among patients younger than 60 years (*n* = 5612) the comorbidity risk was higher for BMI ranging 27.5‒29.9 kg/m^2^ compared with BMI 25.0‒27.4 kg/m^2^ (RR = 1.38; 95% CI 1.27‒1.50 vs. RR = 0.86; 95% CI 0.81‒0.90). In patients older than 60 years (*n*= 3455) the risk was lower for a BMI in the range 27.5–29.9 kg/m^2^ compared with a BMI in the range 25.0–27.4 kg/m^2^ (RR = 1.11; 95% CI 1.05‒1.18 vs. RR = 1.28; 95% CI 1.21‒1.35). These results suggest that being moderately overweight is a marker of a healthy aging process and might protect, at least in part, against comorbidity. However, further research is needed to better understand this unexpected finding.

## 1. Introduction

Comorbidity can be defined, in general, as one or more clinical conditions that has existed or may occur during the clinical course of a patient [1,2] and is usually considered the expression of individual’s fragility that might entail a reduced functional capacity and an impaired quality of life [3]. In developed countries, the burden of comorbidity is becoming heavier in terms of healthcare costs, due to the growing number of patients suffering from multiple illnesses, requiring frequent hospitalizations and prolonged pharmacological treatments [4]. The occurrence of comorbidity is particularly evident among the elderly population: a large-scale study conducted in Scotland reported that while only 30.4% of adults aged 45 to 64 years had at least two chronic conditions, the percentage rose to 64.9% among adults between 65 and 84 years old, even to 80% in those above 85 years old [5]. Similarly, among the US population recruited by the Medicare study, 71.7% of beneficiaries had two or more disorders, and the mean cumulative duration of 19 disease conditions summed up to 23.6 person-years [6]. The latter study drew attention to important issues: (i) the distribution of comorbidity among the elderly is quite heterogeneous, with coexistence of subgroups of subjects showing no or few disease conditions, and others with six or more different conditions and (ii) the healthcare expenditure attributable to comorbidity is proportional to the overall number of illnesses rather than to their cumulative duration. In many populations, elderly patients with comorbidities can even outnumber those suffering from a single illness [7] and some projections suggest that this trend is becoming an alarming phenomenon worldwide. From the above, it is clear the importance of accurately identifying the major determinants of comorbidity and to operate a careful stratification of the individual’s risk to ensure effective preventive measures, especially in subjects who are entering the early stages of the aging process. Several epidemiological studies conducted over the past two decades in various populations suggested that the main factors associated with comorbidity are sex [8], age [9], socioeconomic conditions [10], and body mass index (BMI) [11,12]. With regard to the latter factor, a number of studies have reported an increased risk of frailty and mortality in overweight subjects when compared to control populations with a normal BMI [13,14,15]. However, studies on the relationship between comorbidity and overweight usually focused on a single condition [16] and it is not clear whether it follows the same pattern observed for mortality. The aim of this study was to analyze the comorbidity risk according to BMI categories in a population of Northern Sardinia, Italy.

## 2. Materials and Methods

### 2.1. Study Design and Participants

This was a cross-sectional single-centre study. Medical charts of adult subjects undergoing upper endoscopy were collected and included in a computerized database. Patients referred to the Gastroenterology Section, Department of Internal Medicine, University of Sassari, Italy, were interviewed by a trained gastroenterologist at the time of endoscopy, and the information was recorded in a structured questionnaire as previously described [17]. Data regarding demographic details, marital status, occupation and smoking habits were retrieved. Body mass index was calculated as weight/height^2^ (kg/m^2^). More specifically, body height was measured in centimeters using a stadiometer with the patient’s head aligned to a horizontal plane, and body weight was measured by an electronic scale with an accuracy up to 0.1 kg. Only data from patients with a stable BMI were considered eligible, for instance, patients who had unintentionally lost more than 5 kg of body weight over a period ranging from 6 to 12 months preceding the upper digestive endoscopy were excluded from the analysis. The presence of a definitive diagnosis at the clinical history was collected and classified dichotomously as present/absent according to the Cumulative Illness Rating Scale (CIRS) [18], as follows: cardiovascular, hypertension, blood, respiratory, eyes-ears-nose-throat-larynx, upper gastrointestinal and pancreas; lower gastrointestinal, liver, kidney, urinary, muscle and bone; central nervous system, endocrine, psychiatric, rheumatic and autoimmune disorders; dementia, miscellanea and malignancy. The leading cause for upper endoscopy was not included in the CIRS. Records from patients younger than 18 years or records missing data of associated illness were excluded from the analysis. In the case of multiple esophago-gastro-duodenoscopies for the same patient during the study period, only information from the last procedure was kept for the analysis.

An Institutional Review Board approval was obtained from the local “Comitato Etico ASL n. 1 di Sassari” (Prot N° 2101/CE).

### 2.2. Statistical Analysis

Variables included in the analysis in addition to the BMI were sex, age, residence (urban or rural area), marital status, smoking habits, occupation, hospitalization and comorbidity. More specifically, a BMI ranging 18.5–24.9 kg/m^2^ was defined as normal and ≥ 30 kg/m^2^ as obesity. In order to better define overweight, a BMI between 25.0 and 27.4 kg/m^2^ was arbitrarily considered as mildly overweight and between 27.5 and 29.9 kg/m^2^ as moderately overweight. Marital status was classified as single, married, widowed or divorced patients. For the analysis, patients were considered as never, former and current smokers. The occupations were divided into four categories: (i) graduated professionals; (ii) technicians and administrators (non-graduated); (iii) clerks and salesmen; and (iv) semiskilled and unskilled workers and uneducated shepherds and peasants [19].

Means and standard deviations (SD) were calculated for continuous variables in men and women separately, whereas for categorical variables, the absolute and relative frequencies were calculated. The association between BMI and comorbidity as a response variable was analysed by a multivariable generalized linear regression model with a Poisson distribution and a log link, while controlling simultaneously for several risk factors known to be associated with an increased risk of comorbidity, including age, sex, marital status, provenience, smoking habits, occupation and hospitalization. Relative risks (RRs) and their 95% confidence intervals (CIs) were calculated. All statistical analyses were performed using SPSS statistical software (version 16.0, Chicago, IL, USA). *p* values lower than 0.05 were considered statistically significant.

## 3. Results

Among a total of 9287 patient charts, 220 were excluded because of a body weight loss greater than 5 kg, and the remaining 9067 were eligible for the purpose of the study. The features of participants are shown in Table 1. There were 65.3% women with a mean age similar to men, although there was a statistically significant (*p* < 0.0001) difference in distribution by age decades (Table 1). In general, patients’ residence was roughly half from urban areas and half from rural areas. The majority of participants (62.2%) had a BMI under 25 kg/m^2^, which was more common in men than in women (65.2% vs. 60.6%; *p* < 0.0001), among which, a few subjects had a BMI lower than 18.5 kg/m^2^ (316, 3.5%). Overall, obesity was detected in 7.4% of the study participants, although severe obesity (BMI ≥ 40 kg/m^2^) was almost absent (47 patients, 0.5%). Interestingly, the prevalence of divorce was very low in both sexes (3.6%). Smoking was more common in men (20.5%) than women (14.3%), and the difference was statistically significant (*p* < 0.0001) (Table 1). The proportion of outpatients was preponderant (77.1% of study participants). The most frequent illness recorded was hypertension followed by endocrine and cardiovascular disease. More women suffered from psychiatric disorders as compared with men (10.5% vs 4.9%). The distribution of the other comorbidities and cancer was similar between the two sexes (Table 2).

The demographic and anthropometric features of the study participants according to comorbidity categories are reported in the supplementary material (Table S1).

**Figure 1:**
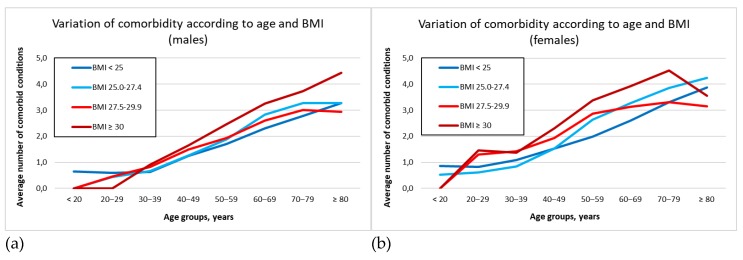
Variation of comorbidity according to age and BMI categories: (**a**) Males; (**b**) Females.

Figure 1 graphically represents the average number of disorders in each BMI category according to the age decade. As expected, in patients younger than 60 years, moderate overweight (BMI between 27.5 and 29.9 kg/m^2^) was clearly associated with a greater burden of illness compared to the mildly overweight category (BMI = 25.0–27.4 kg/m^2^). However, in patients older than 60 years who were moderately overweight (BMI = 27.5‒29.9 kg/m^2^), the number of illnesses was lower than in the mild overweight category, and this was more pronounced in males as compared with females, especially in the 70‒79 year decade (3.47 vs. 4.05). In the supplementary material (Table S1), the variation of comorbidity according to BMI is reported.

**Figure 2:**
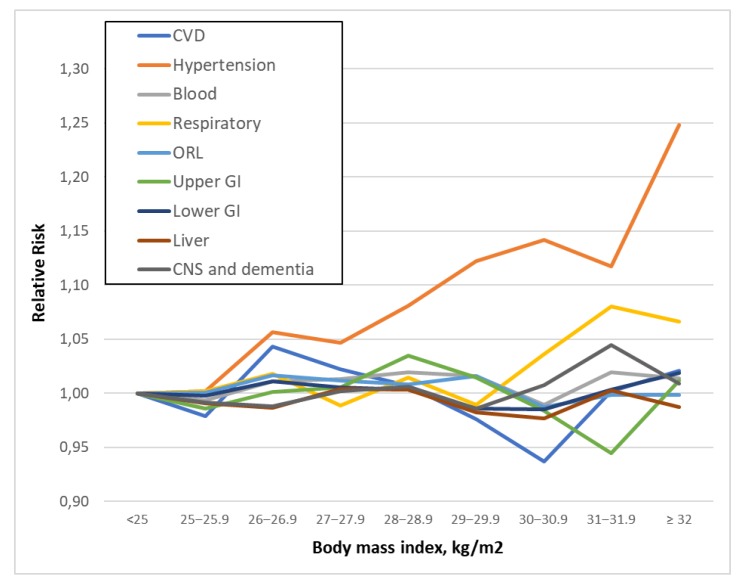
Association of BMI with specific disorder category.

The curves reported in Figure 2 represent the RR for specific illnesses according to weight classification. The majority of patients who were moderately overweight reported a single illness, with the only exception of hypertension, which increased monotonically across all BMI categories (Figure 2).

Table 3 reports the results of the multivariate analysis. After adjusting for potential confounders, the multivariate regression analysis detected an association between comorbidity and age that was statistically significant and progressive. The RR to have a comorbidity was also statistically higher in male patients, those from rural areas and in current or former smokers. Married and widowed patients were at a lower risk compared to single patients, whereas divorced patients were at slightly higher risk. Similarly, a low rank occupation was associated with a significantly increased risk of comorbidity.

**Figure 3:**
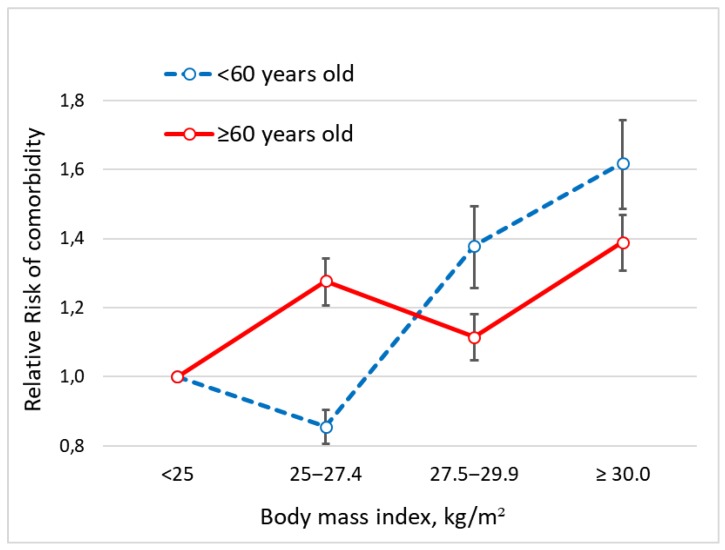
Association of BMI with the risk of comorbidity according to age.

Figure 3 shows RRs for comorbidity according to age and BMI categories. A risk reduction is evident in moderate overweight patients older than sixty compared with younger subjects. More specifically, among patients younger than 60 years (*n* = 5612) the risk of comorbidity was higher for a BMI of 27.5‒29.9 kg/m^2^ compared with a BMI of 25.0‒27.4 kg/m^2^ (RR = 1.38; 95% CI 1.27‒1.50 vs. RR = 0.86; 95% CI 0.81‒0.90). Among patients older than 60 years (*n* = 3,455), the risk for a BMI in the range 27.5–29.9 kg/m^2^ was lower than for a BMI in the range 25.0–27.4 kg/m^2^ (RR = 1.11; 95% CI 1.05‒1.18 vs. RR = 1.28; 95% CI 1.21‒1.15).

## 4. Discussion

Comorbidity, the occurrence of one or more chronic conditions in addition to the patient’s main illness, has become increasingly common in resource-rich populations, especially among people above 65 years of age [20], representing a major challenge for health care systems in these countries [21]. For this reason, there is a growing interest in the study of factors influencing comorbidity, particularly those that are modifiable, including excess weight. Despite several criticisms, BMI remains the most widely used metrics for adiposity due to its simplicity and cheapness [22]. Its popularity is due to the fact that BMI shows a clear relationship with mortality, expressed by a U-shaped curve rising exponentially for increasing values of BMI [23]. Although comorbidity has been related to BMI as well [24], such a relationship has been explored far less frequently than mortality, and it is not clear whether it follows the same pattern observed in the latter.

Our study sought to explore this relationship using a large database of patients undergoing upper gastrointestinal endoscopy, covering a wide age range, and whose anthropometric indices were accurately measured along with the collection of clinical history. The univariate analysis revealed, as expected, the existence of an almost linear relationship between age and the number of co-occurring diseases; nevertheless, a slight levelling off was noted for the moderately overweight BMI category in the oldest patients. These findings were further confirmed by the multivariate analysis after adjusting for potential confounders (i.e., age, sex, marital status, smoking habits, occupation and residence) and effect modifiers (hospitalization), which revealed that compared to subjects with a normal weight or even with subjects mildly overweight, those moderately overweigh suffered a relative lower burden of comorbidity. It is noticeable that this “protective” effect of overweight disappeared for BMIs above 30 kg/m^2^.

Until recently, any increment in BMI above the normal range was thought to entail a net risk for health. The observed increase in comorbidity with excess weight apparently fitted into this conceptual framework and overweight was actually considered a major risk factor for comorbidity [25]. The underlying mechanism explaining this relationship was surmised to be a pro-inflammatory state triggered by visceral adipose tissue favoring the onset of chronic diseases, such as diabetes, cardiovascular disease, neurodegenerative disorders and cancers [26]. However, an increased BMI in elderly people can be the result of the physiological shortening of stature, at least partly, and not necessarily due to the accumulation of adipose tissue [27], with the corollary of a possible overestimation of the impact of BMI on the body’s functioning in the oldest age groups. Hence, the significance of weight gain may vary according to several factors and especially to the different ages in life. For instance, in the young adult, a gain in body weight may reflect an excess of food intake or a lack of physical activity, elements both well known to affect health [28,29]. In contrast, in the elderly who have to face the threat of malnutrition for several reasons (i.e., indigence, tooth loss, dementia, and loneliness, among others), a slight excess of adipose tissue may not be detrimental but paradoxically represent a tool to escape or successfully bypass a negative energy balance. Although this remains the most plausible explanation, it cannot be excluded that the apparent protective effect of overweight could actually be due to reverse causality, namely, subjects displaying a relatively modest comorbidity burden are those whose mechanisms favoring accumulation of adipose tissue are still active, while subjects affected by a greater number of chronic illnesses are those lacking such protective mechanisms. Moreover, since not all illnesses recognize excess weight as a primary causal factor, being overweight could actually lead to an increase only in weight-dependent comorbidity, whereas total comorbidity, which also includes the weight-independent component, may be relatively less affected [30]. Of course, these considerations are speculative and require some support from empirical evidence.

The literature, reviewed in a meta-analysis by Guh et al. [31] on the relationship between BMI and comorbidity, are conflicting. Our findings are consistent with the results of a limited number of studies. Boland et al. reported a reduction of the comorbidity load in overweight patients [32]; however, the majority of studies reported progressively increased comorbidity associated with BMI categories, including moderately overweight. Conversely, in other series, there was no positive or negative effect of the BMI on the number of concurrent diseases [33,34,35]. A follow up study by Stenholm and coll. in Finland reported that obesity predicted frailty among postmenopausal women [15]. Such discrepancies can be partially explained by the different methodology adopted and, likely, to the BMI cut-off chosen to define an overweight state. Moreover, associations between BMI and comorbidity might differ in different samples and settings because of different distributions of potential confounders, whose impact has long been recognized [36]. In addition, studies looking specifically at the relationship between BMI and comorbidity in its entirety in the elderly are lacking, making it difficult to compare our findings with different series results.

Several strengths and limitations exist in the present study that are worthy of mention. First, the BMI was not calculated from self-reported height and weight, as in many similar studies, but accurately measured with the appropriate methodology [37]. Furthermore, the age range of our population was large enough to allow comparison between young and adult individuals. Third, the comorbidity assessment was particularly accurate and exhaustive, since history collection was performed by a well-trained physician and double checked in all records and charts available in patients. Moreover, the study cohort was limited to Northern Sardinia, with a largely similar ethnic composition of Sardinian natives [38].

On the other hand, the retrospective nature of the study is per se a limitation, and the observational design implies a merely descriptive approach. As a result, causation could not be inferred.

## 5. Conclusions

In conclusion, our findings suggest that moderate overweight in older patients, especially females, is associated with a lighter comorbidity load as compared with their peers who have a normal BMI. Further evidence concerning associations between weight status and comorbidity in older patients is needed to determine if healthy weight guidelines should be different for different age groups”.

## Figures and Tables

**Table 1:** Demographic and anthropometric features of the study participants.

Variables	Men	Women	Total	*p*‒Value
Total number of patients in the database	3237 (34.9%)	6050 (65.1%)	9287	-
Number of patients selected for analysis	3143 (34.7%)	5924 (65.3%)	9067	-
Age (years, mean ± SD ^1^)	53.35 ± 16.78	51.74 ± 16.97	52.30 ± 16.93	<0.0001
Age decades (years)				<0.0001
<20	51 (1.6%)	129 (2.2%)	180 (2.0%)	
20‒29	247 (7.9%)	616 (10.4%)	863 (9.5%)	
30‒39	462 (14.7%)	801 (13.5%)	1263 (13.9%)	
40‒49	517 (16.4%)	1045 (17.6%)	1562 (17.2%)	
50‒59	586 (18.6%)	1158 (19.5%)	1744 (19.2%)	
60‒69	686 (21.8%)	1199 (20.2%)	1885 (20.8%)	
70‒79	472 (15.0%)	778 (13.1%)	1250 (13.8%)	
≥80	122 (3.9%)	198 (3.3%)	320 (3.5%)	
Area of residence				0.029
Urban	1504 (47.9%)	2978 (50.3%)	4482 (49.4%)	
Rural	1639 (52.1%)	2946 (49.7%)	4585 (50.6%)	
BMI ^2^ (kg/m^2^)				<0.0001
<25.0	2050 (65.2%)	3592 (60.6%)	5642 (62.2%)	
25.0‒27.4	546 (17.4%)	1450 (24.5%)	1996 (22.0%)	
27.5‒29.9	315 (10.0%)	443 (7.5%)	758 (8.4%)	
≥30	232 (7.4%)	439 (7.4%)	671 (7.4%)	
Marital status				<0.0001
Single	863 (27.5%)	1613 (27.2%)	2476 (27.3%)	
Married	2093 (66.6%)	3491 (58.9%)	5584 (61.6%)	
Widowed	89 (2.8%)	589 (9.9%)	678 (7.5%)	
Divorced	98 (3.1%)	231 (3.9%)	329 (3.6%)	
Smoking habits				< 0.0001
Never smoker	1470 (46.8%)	3335 (56.3%)	4805 (53.0%)	
Current smoker	644 (20.5%)	845 (14.3%)	1489 (16.4%)	
Former smoker	1029 (32.7%)	1744 (29.4%)	2773 (30.6%)	
Occupation ^3^				< 0.0001
Class I	226 (7.2%)	503 (8.5%)	729 (8.0%)	
Class II	953 (30.3%)	1222 (20.6%)	2175 (24.0%)	
Class III	1609 (51.2%)	1030 (17.4%)	2639 (29.1%)	
Class IV	355 (11.3%)	3169 (53.5%)	3524 (38.9%)	

^1^ SD, standard deviation; ^2^ BMI, body mass index; ^3^ Class I: graduated professionals; Class II: technicians and administrators (non-graduated); Class III: clerks and salesmen; Class IV: semiskilled and unskilled workers, and uneducated shepherds and peasants.

**Table 2:** Clinical features of study participants.

Variables	Men	Women	Total
	3143 (34.7%)	5924 (65.3%)	9067
Hospitalization			
Yes	862 (27.4%)	1218 (20.6%)	2080 (22.9%)
No	2281 (72.6%)	4706 (79.4%)	6987 (77.1%)
Illnesses			
Cardiovascular	403 (12.8%)	505 (8.5%)	908 (10.0%)
Hypertension	797 (25.4%)	1486 (25.1%)	2283 (25.2%)
Blood	142 (4.5%)	224 (3.8%)	366 (4.0%)
Respiratory	250 (8.0%)	423 (7.1%)	673 (7.4%)
Eyes, ears, nose, throat, and larynx	85 (2.7%)	162 (2.7%)	247 (2.7%)
Upper GI and pancreas	208 (6.6%)	357 (6.0%)	565 (6.2%)
Lower GI	174 (5.5%)	408 (6.9%)	582 (6.4%)
Liver	361 (11.5%)	418 (7.1%)	779 (8.6%)
Kidney	105 (3.3%)	179 (3.0%)	284 (3.1%)
Urinary	224 (7.1%)	293 (4.9%)	517 (5.7%)
Muscle and bone	161 (5.1%)	720 (12.2%)	881 (9.7%)
Central nervous system	143 (4.5%)	316 (5.3%)	459 (5.1%)
Endocrine	463 (14.7%)	1457 (24.6%)	1920 (21.2%)
Psychiatric disorders and dementia	153 (4.9%)	621 (10.5%)	774 (8.5%)
Rheumatic disorders	183 (5.8%)	733 (12.4%)	916 (10.1%)
Miscellanea	977 (31.1%)	2142(36.2%)	3119 (34.4%)
Malignancy	221 (7.0%)	441 (7.4%)	662 (7.3%)

**Table 3:** Multivariate analysis to evaluate the association between multimorbidity and body mass index, after adjusting for potential confounders.

Variable	RR ^#^ (95% Confidence Interval)	*p*‒Value
Sex		
Female	1	
Male	1.22 (1.18‒1.27)	< 0.0001
Age decades (years)		
<20	1	
20‒29	1.03 (0.85‒1.25)	0.757
30‒39	1.41 (1.17‒1.71)	<0.0001
40‒49	2.40 (1.99‒2.89)	<0.0001
50‒59	3.40 (2.83‒4.10)	<0.0001
60‒69	4.46 (3.71‒5.37)	<0.0001
70‒79	5.15 (4.28‒6.20)	<0.0001
≥80	5.35 (4.41‒6.48)	<0.0001
Area		
Urban	1	
Rural	1.04 (1.01‒1.07)	0.012
Marital status		
Single	1	
Married	0.94 (0.90‒0.98)	0.004
Widowed	0.87 (0.82‒0.93)	<0.0001
Divorced	1.13 (1.04‒1.23)	0.003
Smoking habits		
Never smoker	1	
Current smoker	1.07 (1.03‒1.12)	0.001
Former smoker	1.06 (1.02‒1.09)	0.001
Occupation		
Class I	1	
Class II	1.11 (1.05‒1.19)	0.001
Class III	1.09 (1.02‒1.16)	0.008
Class IV	1.16 (1.09‒1.23)	<0.0001
Hospitalization		
Outpatients	1	
Inpatients	1.44 (1.40‒1.49)	<0.0001
BMI * (kg/m^2^)		
<25.0	1	
25.0‒27.4	1.15 (1.10‒1.19)	<0.0001
27.5‒29.9	1.16 (1.10‒1.22)	<0.0001
≥30.0	1.42 (1.36‒1.49)	<0.0001

^#^ RR, relative risk; * BMI, body mass index.

## Supplementary Materials

The following are available online at https://www.mdpi.com/1660-4601/16/19/3656/s1, Table S1: Demographic and anthropometric features of study participants according to comorbidity category.
