# Supplementary material for: Overweight: A Protective Factor against Comorbidity in the Elderly
Source: Int J Environ Res Public Health. 2019 Sep 29;16(19):3656. doi: 10.3390/ijerph16193656 (PMC6801595; doi:10.3390/ijerph16193656)
Supplement: Supplementary file 1 [file ijerph-16-03656-s001.pdf]

Table S1. Demographic and anthropometric features of study participants according to comorbidity category.

Variables	Number of Conditions in Addition to the Main Illness		
	**None**	**1–2**	**≥3**
Number of patients selected for analysis	2069 (22.8%)	4007 (44.2%)	2991 (33.0%)
Age (years, mean ± SD [1])	39.26 ± 13.81	51.08 ± 15.92	62.95 ± 12.81
Sex			
Female	1244 (60.1%)	2631 (65.7%)	2049 (68.5%)
Male	825 (39.9%)	1376 (34.3%)	942 (31.5%)
Area of residence			
Urban	1082 (52.3%)	1979 (49.4%)	1421 (47.5%)
Rural	987 (47.7%)	2028 (50.6%)	1570 (52.5%)
Marital status			
Single	892 (43.1%)	1093 (27.3%)	491 (16.4%)
Married	1086 (52.5%)	2507 (62.5%)	1991 (66.6%)
Widowed	43 (2.1%)	255 (6.4%)	380 (12.7%)
Divorced	48 (2.3%)	152 (3.8%)	129 (4.3%)
Smoking habits			
Never smoker	1103 (53.3%)	2140 (53.4%)	1562 (52.2%)
Current smoker	289 (14.0%)	640 (16.0%)	560 (18.7%)
Former smoker	677 (32.7%)	1227 (30.6%)	869 (29.1%)
Hospitalization			
Outpatients	1877 (90.7%)	3179 (79.3%)	1931 (64.6%)
Inpatients	192 (9.3%)	828 (20.7%)	1060 (35.4%)
BMI [2] (kg/m^2)			
< 25.0	1278 (61.8%)	2631 (65.7%)	1733 (58.0%)
25.0–27.4	629 (30.4%)	808 (20.1%)	559 (18.7%)
27.5–29.9	94 (4.5%)	340 (8.5%)	324 (10.8%)
≥ 30.0	68 (3.3%)	228 (5.7%)	375 (12.5%)

SD [1], standard deviation, BMI [2], body mass index.
